# The Utility of Predicting Hospitalizations Among Patients With Heart Failure Using mHealth: Observational Study

**DOI:** 10.2196/18496

**Published:** 2020-12-22

**Authors:** Susie Cartledge, Ralph Maddison, Sara Vogrin, Roman Falls, Odgerel Tumur, Ingrid Hopper, Christopher Neil

**Affiliations:** 1 College of Nursing and Health Sciences Flinders University Adelaide Australia; 2 Institution for Physical Activity and Nutrition School of Exercise and Nutrition Sciences Deakin University Geelong Australia; 3 Department of Medicine Western Health University of Melbourne Melbourne Australia; 4 Department of Epidemiology and Preventive Medicine Monash University Melbourne Australia; 5 Western Health Chronic Disease Alliance Melbourne Australia

**Keywords:** cardiac failure, heart failure, readmission, hospitalization, risk prediction, mHealth

## Abstract

**Background:**

Heart failure decompensation is a major driver of hospitalizations and represents a significant burden to the health care system. Identifying those at greatest risk of admission can allow for targeted interventions to reduce this risk.

**Objective:**

This paper aims to compare the predictive value of objective and subjective heart failure respiratory symptoms on imminent heart failure decompensation and subsequent hospitalization within a 30-day period.

**Methods:**

A prospective observational pilot study was conducted. People living at home with heart failure were recruited from a single-center heart failure outpatient clinic. Objective (blood pressure, heart rate, weight, B-type natriuretic peptide) and subjective (4 heart failure respiratory symptoms scored for severity on a 5-point Likert scale) data were collected twice weekly for a 30-day period.

**Results:**

A total of 29 participants (median age 79 years; 18/29, 62% men) completed the study. During the study period, 10 of the 29 participants (34%) were hospitalized as a result of heart failure. For objective data, only heart rate exhibited a between-group difference. However, it was nonsignificant for variability (*P*=.71). Subjective symptom scores provided better prediction. Specifically, the highest precision of heart failure hospitalization was observed when patients with heart failure experienced severe dyspnea, orthopnea, and bendopnea on any given day (area under the curve of 0.77; sensitivity of 83%; specificity of 73%).

**Conclusions:**

The use of subjective respiratory symptom reporting on a 5-point Likert scale may facilitate a simple and low-cost method of predicting heart failure decompensation and imminent hospitalization. Serial collection of symptom data could be augmented using ecological momentary assessment of self-reported symptoms within a mobile health monitoring strategy for patients at high risk for heart failure decompensation.

## Introduction

Heart failure (HF) is a complex, chronic, and debilitating cardiac condition currently estimated to affect 38 million people internationally [1]. In Australia, 2014 prevalence estimates indicated that there were 480,000 adults living with HF, which represents 2.1% of the Australian population, with the prevalence forecast to significantly increase [2]. HF is caused by the inability of the heart to fill and eject sufficient blood to meet bodily demands, resulting in symptoms such as dyspnea, fatigue, and palpitations [3]. The exacerbation of HF symptoms, representing HF decompensation, is a major driver of hospitalization rates. HF hospitalizations represent a significant proportion of the total expenditure for HF in Australia annually [2]. Therefore, monitoring HF symptoms is essential in order to identify and prevent potential HF decompensation and subsequent hospitalization.

Collaboration between people living with HF and health care professionals (eg, heart failure nurses) is critical for monitoring HF symptoms and potential exacerbations [4]. While there are smartphone apps that focus on symptom monitoring, none currently provide risk prediction [4]. While HF mortality can be predicted with reasonable accuracy [5,6], risk prediction for HF hospitalization has demonstrated only modest performance in models reported to date. Variables used in HF risk predication models have included, in isolation or in combination [7], administrative data (such as Medicare claims data) [8], patient characteristics, clinical data, and geomapping [9]. However, the quantitation of self-reported subjective symptoms as an early indication of decline and therefore risk has on the whole been overlooked. Therefore, the aim of this study was to compare the predictive value of objectively and subjectively measured HF respiratory symptoms on imminent HF decompensation and subsequent hospitalization within a 30-day period.

## Methods

### Study Design, Setting, and Participants

We conducted a prospective observational pilot study with participants identified via cardiologist assessment as being at high risk of a HF hospitalization. Participants were recruited from a single-center HF outpatient clinic within a tertiary hospital in Melbourne, Australia. Eligible participants were older than 18 years, had a physician-documented HF diagnosis, had a previous hospital admission for HF exacerbation, were on maximum tolerated pharmacotherapy, and were able to read and understand English. Exclusion criteria included severe HF symptoms (New York Heart Association Class IV), advanced malignancy, cognitive impairment, and use of end-of-life care. This study was approved by the Western Health Human Research Ethics Committee (2016.071).

### Measures

Participants were visited twice weekly by a research assistant (one a biomedical science graduate the other a medical doctor) for a 30-day period from June 2016 to May 2017. The research assistants collected all measurements, including the subjective respiratory scores, from patients. Study data and sources are described in Table 1.

**Table 1:** Variables collected during study period and associated data sources.

Variables			Data source
**Objective measures**			
	Blood pressure	Validated study sphygmomanometer	
	Heart rate	Manual pulse	
	Weight	Validated study scales	
	B-type natriuretic peptide	Point-of-care testing	
**Subjective measures**			
	Dyspnea	5-point Likert scale^a^	
	Bendopnea	5-point Likert scale^a^	
	Orthopnea	5-point Likert scale^a^	
	Paroxysmal nocturnal dyspnea	5-point Likert scale^a^	
**Other variables**			
	Demographics	Medical record and participant survey	
	Medical history	Medical record	
	Hospitalization status	Medical record	

^a^1 indicates no symptoms and 5 indicates severe symptoms.

Subjective symptoms of dyspnea, orthopnea, bendopnea, and paroxysmal nocturnal dyspnea (PND) were chosen because they are routinely used indicators of clinical status in HF, each of which feature in 2 key diagnosis criteria [10,11]. Likert scales to instantaneously quantify dyspnea in HF populations have been researched using 7- and 5-point scales [12,13]. Additionally, the 5-point Likert scale–quantified dyspnea has previously demonstrated a relationship with subsequent emergency readmission [13].

### Statistical Analysis

Given that this is a pilot study, no formal power calculations were undertaken. Baseline characteristics are presented as median (interquartile range) or frequency (percentage) and are compared between hospitalized and nonhospitalized patients using a rank sum test and Fisher exact test.

To assess whether objective measures (blood pressure, heart rate [HR], variation in HR, weight, and B-type natriuretic peptide [BNP]) were associated with hospitalization, their mean value, standard deviation, and slope of change were calculated over 7 days prior to hospitalization (for admitted patients) and over the whole observation period for others (with at least 7 days’ clearance before and after any hospitalization). These were then compared using a rank sum test. The same technique was applied using a symptom severity score, and their variability was compared by calculating a median score and range of scores.

To determine the optimal severity cutoff value on the Likert scale for each respective symptom, a random day within the 7 days prior to hospitalization (for hospitalized patients) and any random day for others was chosen. The area under the receiver operating characteristics curve (AUC) and the Youden index (YI) were calculated. This process was repeated 1000 times with different combinations of random days. The cutoff value with the most frequent highest AUC and Youden index was chosen. This cutoff was then used for all analyses.

To determine which combination of symptoms best predicted hospitalization, we calculated AUC, YI, sensitivity, and specificity and compared them among all combinations of symptoms (eg, bendopnea and orthopnea, bendopnea and dyspnea, bendopnea and dyspnea and orthopnea—a total of 10 possible combinations) on 1000 combinations of randomly chosen days (as described above). In the next step, 2 random consecutive symptom measurements (usually 2 to 3 days apart) were chosen, and we determined the ability to predict hospitalization if the symptom was severe on either day or both days or if the symptom severity increased across the 2 days. This was performed separately for each symptom and for all 10 combinations of symptoms (as described above). Sensitivity, specificity, AUC, and YI were calculated. All analyses were performed using Stata 15.1 (StataCorp).

## Results

### Baseline Population Characteristics

A total of 30 participants met the study inclusion criteria and provided written informed consent; however, one participant withdrew from the study shortly after enrollment. During the study, 10 of the 29 participants (34%) were hospitalized as a result of decompensated HF, as adjudicated by the Boston criteria on file review (the comparator group). Another participant was admitted for infection without congestion.

As demonstrated in Table 2, the participants who were hospitalized had higher left ventricular ejection fractions but worse baseline Minnesota Living With HF scores and were on less angiotensin- converting enzyme inhibitors and angiotensin II receptor blocker therapies.

**Table 2:** Baseline demographics and comparison by heart failure hospitalization.

Characteristic		All participants (N=29)	Not hospitalized (19/29, 66%)	Hospitalized (10/29, 34%)	*P* value
Age (years), median (IQR)		79 (69-84)	74 (68-82)	81.5 (73-89)	.12
**Sex, n (%)**					**.69**
	Male	18 (62)	11 (58)	7 (70)	
	Female	11 (38)	8 (42)	3 (30)	
**Ethnic group, n (%)**					**.56**
	Non-Indigenous Australian	7 (24)	6 (32)	1 (10)	
	European	8 (28)	5 (26)	3 (30)	
	Indian	1 (3)	1 (5)	0 (0)	
	Pacific Islander	5 (17)	2 (11)	3 (30)	
	Other	8 (28)	5 (26)	3 (30)	
Born in Australia, n (%)		11 (39)	9 (47)	2 (22)	.25
**Education, n (%)**					**.12**
	Some high school	19 (66)	15 (79)	4 (40)	
	Trade certificate	8 (28)	4 (21)	4 (40)	
	Some university	2 (7)	0 (0)	2 (20)	
**Work status, n (%)**					**.69**
	Part-time	1 (3)	1 (5)	0 (0)	
	Full-time	2 (7)	2 (11)	0 (0)	
	Retired	26 (90)	16 (84)	10 (100)	
**Household income (Aus $)^a^, n (%)**					**.40**
	<20,000	18 (62)	10 (53)	8 (80)	
	20,000-30,000	10 (34)	8 (42)	2 (20)	
	30,000-40,000	1 (3)	1 (5)	0 (0)	
**Years since HF^b^ diagnosis, n (%)**					**.42**
	≤5 years	18 (62)	13 (68)	5 (50)	
	5-10 years	4 (14)	3 (16)	1 (10)	
	10-20 years	5 (17)	2 (11)	3 (30)	
	>20 years	2 (7)	1 (5)	1 (10)	
HFrEF^c^, n (%)		16 (55)	12 (63)	4 (40)	.27
**Baseline NYHA^d^, n (%)**					**.09**
	NYHA II	10 (34)	9 (47)	1 (10)	
	NYHA III	19 (66)	10 (53)	9 (90)	
LVEF^e^, median (IQR)		35 (28-50)	30 (22-46)	50 (35-57)	.02
Baseline Minnesota Living With HF score^f^, median (IQR)		58 (48-70)	53 (40-62)	72.5 (54-81)	.02
HF hospitalization in previous 6 months, n (%)		25 (86)	17 (89)	8 (80)	.59
Emergency department attendance in previous 12 months, n (%)		21 (75)	13 (68)	8 (89)	.37
Body mass index, median (IQR)		30.42 (25.65-38.72)	30.80 (25.65-39.82)	30.27 (25.13-32.25)	.65
**Medical history, n (%)**					
	Hypertension	27 (93)	17 (89)	10 (100)	.53
	Ischemic heart disease	16 (55)	10 (53)	6 (60)	>.99
	Hypercholesterolemia	22 (76)	16 (84)	6 (60)	.19
	Atrial fibrillation	19 (66)	11 (58)	8 (80)	.41
	Cerebrovascular accident	7 (24)	6 (32)	1 (10)	.37
	Diabetes mellitus	17 (59)	10 (53)	7 (70)	.45
	COPD^g^ or asthma	11 (38)	6 (32)	5 (50)	.43
**Use of therapies**					
	Loop diuretic, n (%)	27 (93)	17 (89)	10 (100)	.53
	Daily loop diuretic dose, median (IQR)	80 (80-120)	80 (40-80)	100 (80-60)	.09
	β blocker, n (%)	26 (90)	17 (89)	9 (90)	>.99
	ACE-I^h^ or ARB^i^, n (%)	22 (76)	17 (89)	5 (50)	.03
	Aldosterone antagonist, n (%)	12 (41)	8 (42)	4 (40)	>.99

^a^At the time of publication, a currency exchange rate of Aus $1=US $0.74 was applicable.

^b^HF: heart failure.

^c^HFrEF: heart failure with reduced ejection fraction.

^d^NYHA: New York Heart Association.

^e^LVEF: left ventricular ejection fraction.

^f^The Minnesota Living With Heart Failure scoring range is from 0 to 105, with higher scores indicating poorer health-related quality of life.

^g^COPD: chronic obstructive pulmonary disease.

^h^ACE-I: angiotensin-converting enzyme inhibitor.

^i^ARB: angiotensin II receptor blocker.

### Objective Measures

Of the 4 objective measures collected, only HR demonstrated a significant difference between groups (median 80 vs 67 beats per minute [bpm]; *P*=.02) (Table 3). Variation in HR over the study period, however, was nonsignificant between groups (7 vs 6 bpm; *P*=.71). Participants admitted to the hospital demonstrated higher BN*P* values (median 1113 vs 546 pg/mL; *P*=.09), with a higher average daily increase prior to hospitalization (20 vs 0.05 pg/mL/d; *P*=.08).

**Table 3:** Objective and subjective measures.

Measures				Not hospitalized, median (IQR), (n=19)		Hospitalized, median (IQR), (n=10)		*P* value
**Objective measures**								
	**Weight**							
		Average (kg)	87 (73.8 to 95.6)		84.5 (64 to 96.1)		.52	
		Variability (standard deviation) (kg)	0.97 (0.63 to 1.30)		1.07 (0.61 to 2.86)		.57	
		Slope (kg/d)	0.00 (–0.04 to 0.02)		0.13 (–0.15 to 0.33)		.26	
	**Systolic blood pressure**							
		Average (mmHg)	121.8 (108 to 128.7)		123.2 (113.8 to 131.7)		.65	
		Variability (standard deviation) (mmHg)	12.8 (8.3 to 15.4)		15.4 (11.1 to 17.6)		.29	
		Slope (mmHg/d)	0.25 (–0.37 to 0.64)		1.06 (–0.82 to 10.5)		.31	
	**Heart rate**							
		Average (bpm^a^)	67.4 (62.0 to 76.2)		80.4 (74.3 to 88.0)		.02	
		Variability (standard deviation) (bpm)	7.5 (4.3 to 10.0)		7.2 (3.6 to 14.4)		.71	
		Slope (bpm/d)	–0.04 (–0.17 to 0.36)		–0.61 (–1.59 to 0.87)		.38	
	**B-type natriuretic peptide**							
		Average (pg/mL)	545.6 (237.4 to 891.7)		1112.5 (554.5 to 1565)		.09	
		Variability (standard deviation) (pg/mL)	99.9 (47.5 to 231.9)		164.4 (75.2 to 186.71)		.83	
		Slope (pg/mL/d)	0.05 (–4.85 to 5.81)		19.65 (4 to 41.45)		.08	
**Subjective measures^b^**								
	**Dyspnea**							
		Median symptom scores	2 (1 to 3)		4 (3 to 4)		.009	
		Ranges in symptom score	2 (1 to 3)		0 (0 to 1)		.003	
	**Orthopnea**							
		Median symptom scores	1 (1 to 2)		3.3 (2 to 4)		.007	
		Ranges in symptom score	1 (0 to 2)		0.5 (0 to 1)		.22	
	**Bendopnea**							
		Median symptom scores	2 (1 to 3)		4 (3.5 to 5)		.007	
		Ranges in symptom score	2 (1 to 3)		0 (0 to 1)		.006	
	**Paroxysmal nocturnal dyspnea**							
		Median symptom scores	1 (1 to 2)		3 (1.5 to 4)		.009	
		Ranges in symptom score	1 (1 to 3)		0 (0 to 1)		.004	

^a^bpm: beats per minute.

^b^For subjective measures, the median is the median and IQR of the participants’ median scores. Range is the median and IQR of participants’ range of scores; for example, if the range was 1, that patient had a symptom of 3 and 4 only.

### Subjective Measures

**Figure 1:**
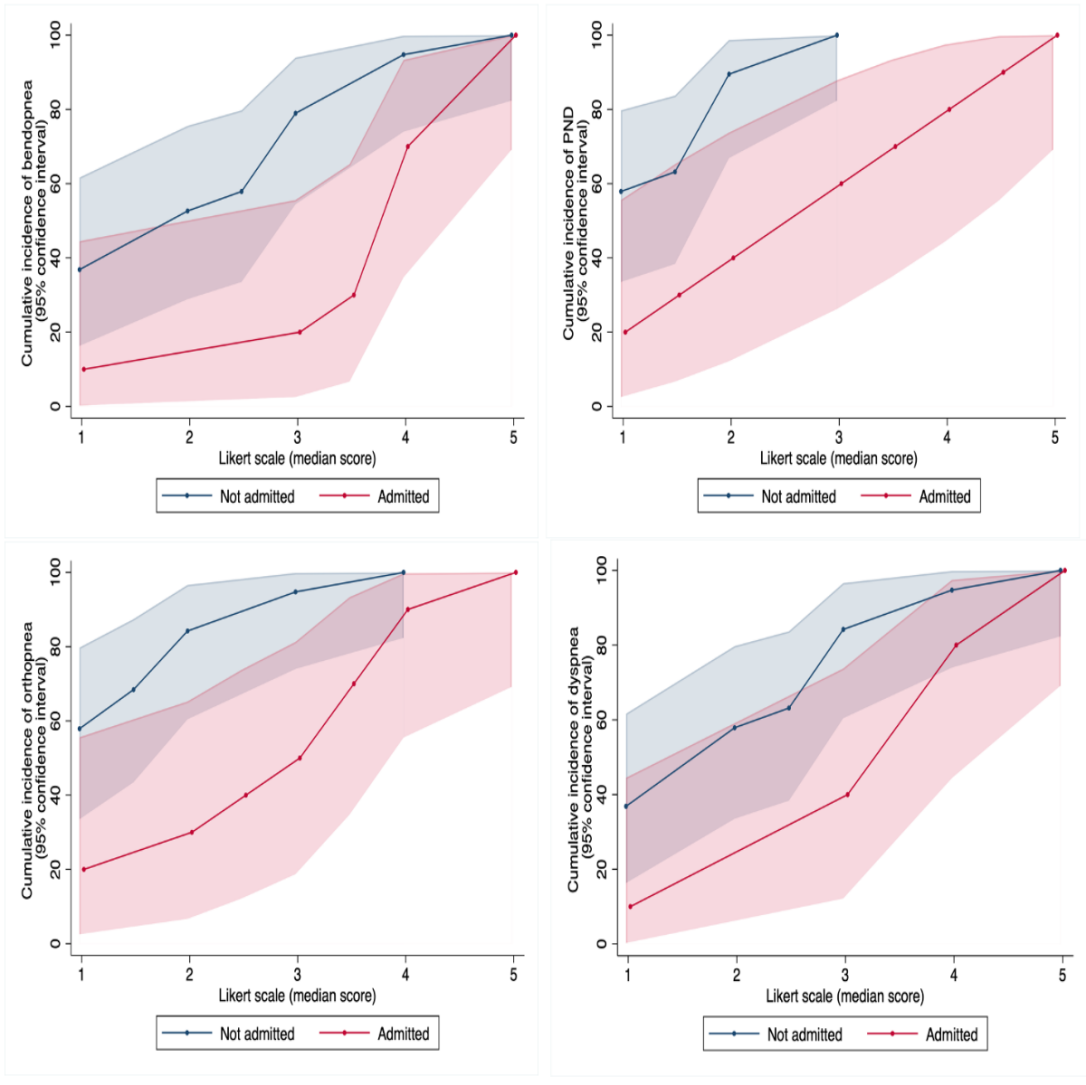
Subjective symptom measurements experienced at each severity level of the Likert scale, showing the weighting of severity in the hospitalized versus nonhospitalized group.

**Figure 2:**
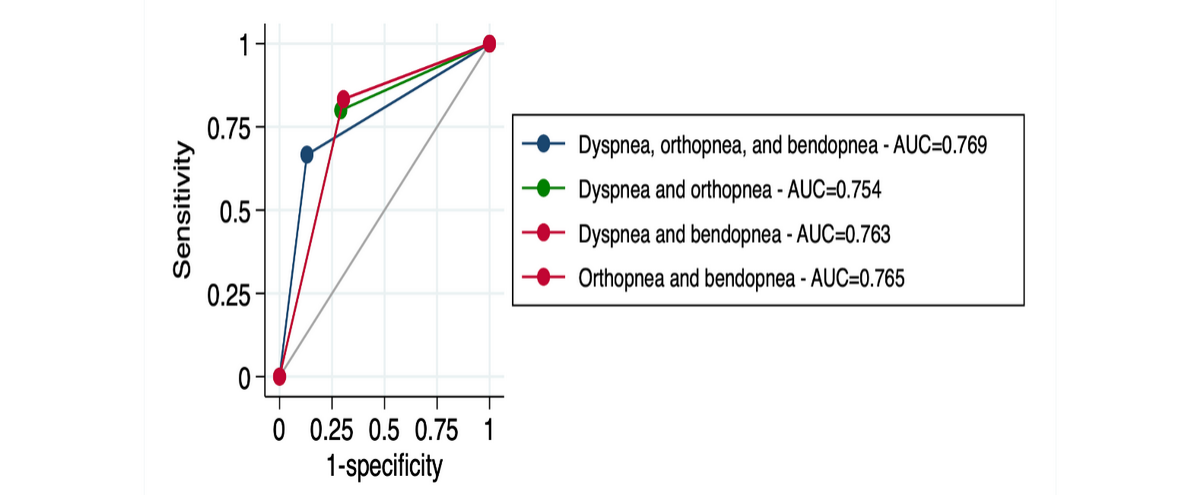
Areas under the curve for subjective respiratory symptoms. AUC: area under the curve.

Symptom scores were examined, with hospitalized patients reporting higher median dyspnea, bendopnea, and PND with a lower 7-day range, implying consistently worse symptoms (all *P*<.01) (Table 3). Figure 1 shows the symptom measurements experienced at each severity level of the Likert scale. Orthopnea was worse in the HF hospitalization group (*P*=.01) but had similar variability (*P*=.22). A symptom score of at least 3 for dyspnea, 2 for orthopnea, and 4 for PND and bendopnea produced the highest AUC and YI for predicting HF hospitalization. Figure 2 shows the areas under the curve for respiratory symptoms.

The highest YI (0.54) and AUC (0.77) were observed when on any given day dyspnea, orthopnea, and bendopnea symptoms were severe. This combination predicted hospitalization with a sensitivity of 81% and a specificity of 73%. Similar results were observed when at least orthopnea and bendopnea were severe (YI=0.53; AUC=0.76; sensitivity of 81%; specificity of 73%). Higher sensitivities but lower specificities were observed when dyspnea and bendopnea (84% and 68%, respectively) or dyspnea and orthopnea (87% and 64%, respectively) were severe (see Multimedia Appendix 1 for graphical representation). All other combinations resulted in a YI <0.50 and an AUC <0.75.

## Discussion

### Principal Findings

In this analysis of the prediction of decompensation and HF hospitalization within 30 days, we demonstrated that compared with objective measures, a simplified system for quantifying respiratory symptom status may be an accurate and useful predictor. Specifically, the highest precision of HF hospitalization was observed when patients with HF experienced severe dyspnea, orthopnea, and bendopnea on any given day (AUC=0.77; sensitivity of 83%; specificity of 73%). Early detection of deterioration would allow the care team to provide agile HF care that may be able to prevent subsequent hospital admission.

The lack of a sound risk prediction tool for imminent HF hospitalization makes organization and prioritization of HF care challenging [5]. Current Australian HF guidelines call for systems of care with an “alert system to flag patients who are displaying signs of clinical deterioration and pathways for rapid medical review” [3]. Given that subjective data outperformed objective data in this pilot cohort, there are opportunities for patients to be able to regularly log respiratory symptoms. This eliminates the need for patients to regularly use medical equipment (eg, sphygmomanometer, scales) to collect prediction data.

Real-time regular data collection of a participant’s state, such as HF symptoms, can be conducted through ecological momentary assessment (EMA) [14]. This method allows a picture to be formed of a participant’s symptoms, reducing recall bias. EMA data are best collected by electronic means, such as mobile and wireless devices (mobile health [mHealth]), to ensure easy, timely, and compliant documentation [14,15].

The ease of collection of EMA self-reported respiratory symptom data via mHealth could lead to large data sets for analysis. Artificial intelligence and machine learning are increasingly being used to provide clinically meaningful predictive data analysis, especially with large data sets [16]. This technique can be applied to build automated clinical decision systems for problems such as hospitalization risk [16].

### Limitations

The main limitation of this pilot study is its sample size and length of follow-up. The small sample size may affect the variability of some measures (eg, weight), and this should be addressed in future larger trials over a longer period. Future studies could establish baseline respiratory symptoms for patients and examine the timeline of changes in the severity of symptoms and of hospitalization. Additionally, there was selection bias, as patients were recruited from a single-center HF clinic, and this may not represent a typical HF population.

However, unlike previous studies using patients drawn from clinical trials [17], our patients were recruited from standard practice, which may increase the generalizability of the study. Another strength is the use of primary data collected during the study rather than secondary data, such as trial databases, registries, and administrative claims data, which have been used in other studies.

### Conclusions

The use of patient-reported serial quantification of 4 key respiratory HF symptoms (dyspnea, bendopnea, orthopnea, and PND) may provide low-cost detection of imminent decompensation and therefore potential hospitalization. Future research should focus on testing and validating this model with a larger sample, augmenting the findings using an EMA of self-reported HF symptoms via mHealth, and using artificial intelligence data analysis techniques to increase risk prediction accuracy.

## Appendix

Multimedia Appendix 1 Supplementary figures.

## Abbreviations

AUC: area under the receiver operating characteristics curve
BNP: B-type natriuretic peptide
bpm: beats per minute
EMA: ecological momentary assessment
HF: heart failure
HR: heart rate
mHealth: mobile health
PND: paroxysmal nocturnal dyspnea
YI: Youden index
