# Supplementary material for: The Utility of Predicting Hospitalizations Among Patients With Heart Failure Using mHealth: Observational Study
Source: JMIR Mhealth Uhealth. 2020 Dec 22;8(12):e18496. doi: 10.2196/18496 (PMC7785406; doi:10.2196/18496)
Supplement: Multimedia Appendix 1 [file mhealth_v8i12e18496_app1.docx]

## Supplemental figures


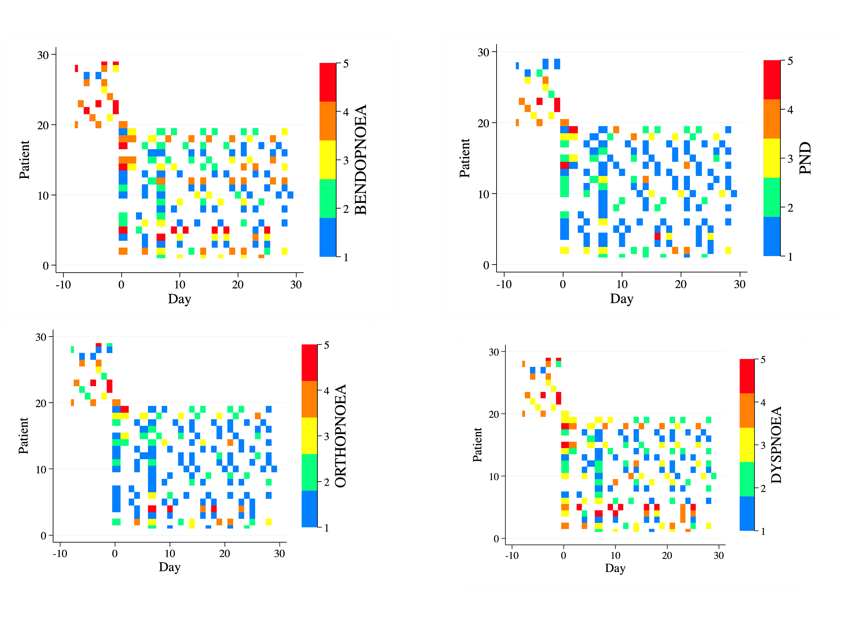
**Supplemental Figure 1.** Heat maps of subjective measurements of both heart failure hospitalised and non-hospitalised patients. Subjective measurements were quantified on a 5-point Likert scale. Day 0 represents day of hospitalisation for hospitalised patients and day of enrolment for non-hospitalised patients. One row represents symptoms of one patients over time. Each square represents patient’s symptom severity on that day (ranging from blue – least severe - to red – most severe). Patients that were hospitalised are presented in top left corner, with x axis representing number of days prior to hospital admission.


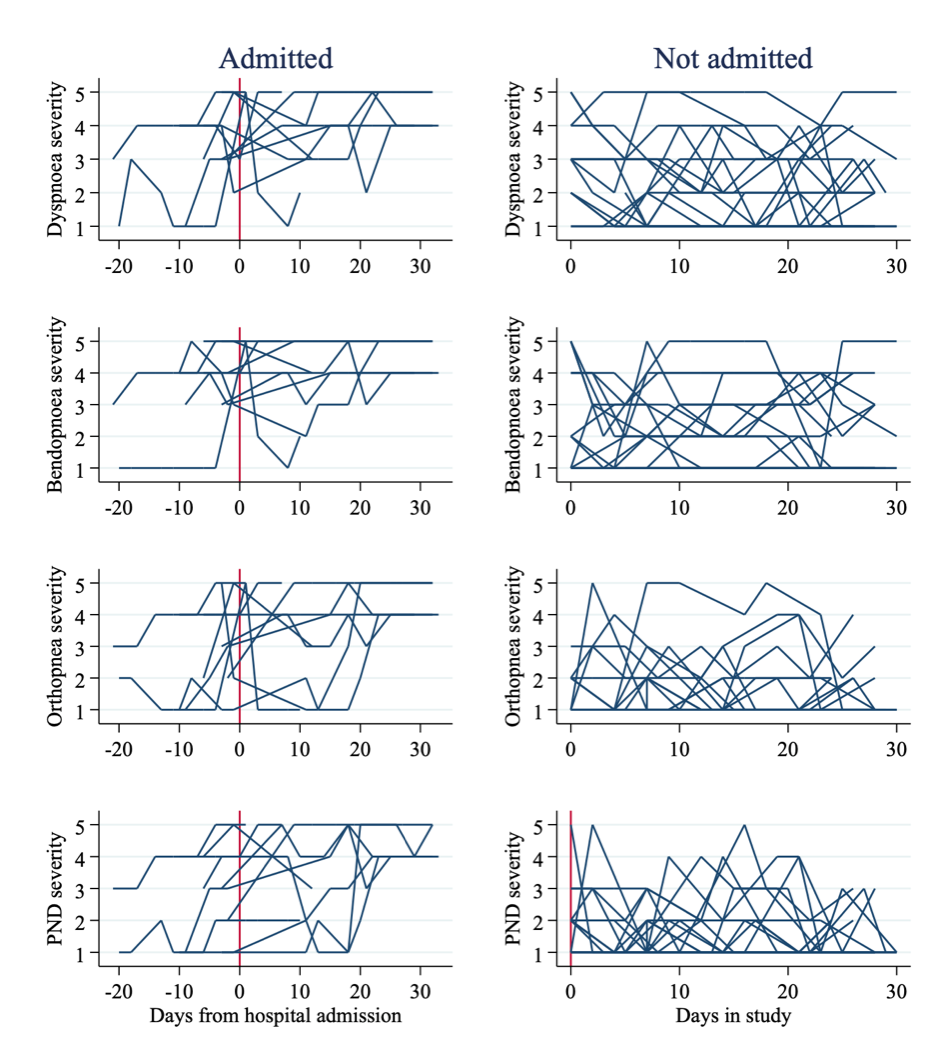


**Supplemental Figure 2.** A comparison of hospitalised to non-hospitalised patients and the tracking of their subjective respiratory symptoms over the study period. Each line represents symptoms over time for one patient.
